# The conversion of formate into purines stimulates mTORC1 leading to CAD-dependent activation of pyrimidine synthesis

**DOI:** 10.1186/s40170-020-00228-3

**Published:** 2020-09-21

**Authors:** Jacqueline Tait-Mulder, Kelly Hodge, David Sumpton, Sara Zanivan, Alexei Vazquez

**Affiliations:** 1 Cancer Research UK Beatson Institute, Switchback Road, Bearsden, Glasgow, G61 1BD UK; 2 Institute of Cancer Sciences, University of Glasgow, Glasgow, UK

## Abstract

**Background:**

Mitochondrial serine catabolism to formate induces a metabolic switch to a hypermetabolic state with high rates of glycolysis, purine synthesis and pyrimidine synthesis. While formate is a purine precursor, it is not clear how formate induces pyrimidine synthesis.

**Methods:**

Here we combine phospho-proteome and metabolic profiling to determine how formate induces pyrimidine synthesis.

**Results:**

We discover that formate induces phosphorylation of carbamoyl phosphate synthetase (CAD), which is known to increase CAD enzymatic activity. Mechanistically, formate induces mechanistic target of rapamycin complex 1 (mTORC1) activity as quantified by phosphorylation of its targets S6, 4E-BP1, S6K1 and CAD. Treatment with the allosteric mTORC1 inhibitor rapamycin abrogates CAD phosphorylation and pyrimidine synthesis induced by formate. Furthermore, we show that the formate-dependent induction of mTOR signalling and CAD phosphorylation is dependent on an increase in purine synthesis.

**Conclusions:**

We conclude that formate activates mTORC1 and induces pyrimidine synthesis via the mTORC1-dependent phosphorylation of CAD.

## Background

Cells activate metabolic pathways during differentiation and de-differentiation. The activation of one or more biochemical reactions can, in turn, trigger a cascade of metabolic changes leading to a distinct metabolic state. We have recently shown that the mitochondrial-dependent oxidation of the third carbon of serine to formate triggers a metabolic switch [1]. This switch is characterized by an increase in metabolic fluxes associated with glycolysis, purine synthesis and pyrimidine synthesis.

Nevertheless, how formate induces pyrimidine synthesis is yet unclear. The formate-dependent induction of pyrimidine synthesis is characterized by a dramatic increase of dihydroorotate levels [1]. Dihydroorotate is the product of carbamoyl phosphate synthetase (CAD), the first step of pyrimidine synthesis. We hypothesized that the formate-dependent increase in adenosine triphosphate (ATP) levels stimulate the enzymatic activity of cytosolic CAD. Mammalian CAD has a half saturation constant for ATP in the mM range [2], which is the range of intracellular ATP levels. However, a theoretical analysis of the full range of behavior indicates that increased ATP is not sufficient to explain the dramatic increase in dihydroorotate levels. A necessary condition is that the maximum CAD activity exceeds the maximum enzymatic activity of a downstream reaction in the pyrimidine synthesis pathway.

CAD maximum activity is regulated by a mechanistic target of rapamycin complex 1 (mTORC1)-dependent phosphorylation [3, 4]. In this way, mTORC1 increases the pyrimidine pool required for cell growth and proliferation. Mechanistically, the mTORC1 kinase complex phosphorylates and activates the ribosomal protein S6 kinase 1 (S6K1), which in turn phosphorylates CAD at Ser 1859. Finally, CAD-Ser1859 phosphorylation increases the maximum CAD activity and consequently pyrimidine synthesis. Here, we demonstrate that formate induces pyrimidine synthesis through the activation of mTORC1 signalling.

## Methods

### Chemicals

Cell culture medium and supplements were obtained from the Life Technologies, and all chemicals were purchased from Sigma-Aldrich unless specified otherwise.

### Cell lines and cultures

HAP1-WT were cultured in IMDM medium supplemented with 10% FBS. HAP1-ΔSHMT2 cells were cultured in the same media supplemented with a mix of 16 μM thymidine and 100 μM hypoxanthine (HT). MDA-MB-231-ΔSHMT2 cells [1] were cultured in DMEM medium supplemented with 10% FBS, glutamine and HT. HT was omitted when seeding cells for experiments. Cell counts and volumes were assessed using the Casy Technology (Innovatis).

### Phosphoproteomics sample preparation

HAP1-ΔSHMT2 cells were seeded in 15 cm dishes and stimulated the following day with 1 mM formate, in the absence of presence of 50 nM rapamycin as indicated. Cells were washed twice in PBS, scraped and lysed in thiourea/urea buffer (6 M urea, 2 M thiourea, 50 mM TRIS pH8.5, 75 mM NaCl) containing complete phosphatase and protease inhibitors. Lysates were sonicated and centrifuged at 16 k G for 5 min at room temperature. Sample supernatants were collected, and the protein concentration measured using the Bradford method. Samples were then snap-frozen in liquid nitrogen and stored at – 80 °C until further analysis.

### Phosphoproteome analysis

Cell lysates were reduced with 10 mM DTT and subsequently alkylated with 55 mM Iodoacetamide. Alkylated proteins were then digested first using Endoproteinase Lys-C (Alpha Laboratories) for 1 h followed by trypsin (Promega) overnight. Peptides were then desalted using C18 SepPak (Waters) filtration and resuspended in a buffer containing 80% acetonitrile (ACN) and 6% trifluoroacetic acid (TFA). Enrichment of phosphorylated peptides was performed using TiO_2_ beads (GL Sciences) and eluted from beads with a buffer 15% NH_4_OH and 40% ACN. Peptides were separated on a nanoscale C_18_ reverse-phase liquid chromatography (20 cm fused silica emitter (New Objective) packed in-house with ReproSil-Pur C18-AQ, 1.9 μm resin (Dr. Maisch)) performed on an EASY-nLC 1200 (Thermo Scientific) coupled online to an Orbitrap Fusion Lumos mass spectrometer (Thermo Scientific). MS data were acquired using the XCalibur software (Thermo Scientific).

The MS raw files were analysed with the MaxQuant computational platform [5] and searched against the human UniProt database using the Andromeda search engine [6]. Trypsin with full-enzyme specificity and maximum two missed cleavages were allowed. Only peptides longer than six amino acids were analysed. Oxidation (Met) and N-acetylation were set as variable modifications, as well as phospho(STY). Carbamidomethylation (Cys) was set as fixed modification. A 1% false discovery rate (FDR) was used for peptides, proteins and phosphopeptide identification.

MaxQuant output Phospho(STY)site.txt file was processed with Perseus software [7]. Reverse and potential contaminant flagged proteins were removed, and only class I sites (= sites accurately localized with localization probability > 0.75 and score difference > 5) were used for the analysis. Intensity values were used for phosphorylation site quantification.

### Metabolite extraction

Metabolite extraction and analysis were performed as previously described [1]. Briefly, HAP1-ΔSHMT2 cells were seeded into 12-well plates at 100.000 cells/well and treated the following day as indicated. For ^15^N tracing, [^15^N-amide]-glutamine was added to the media at 4 mM. To harvest, wells were washed with ice-cold PBS and scraped and extracted with freezer cold extraction solvent (acetonitrile/MeOH/H_2_O (30/50/20)). The extracts were transferred into tubes and centrifuged for 5 min at 18 k G. Supernatants were transferred to LC-MS glass vials and kept at – 80 °C until LC-MS analysis using a pHILIC chromatography and a Q-Exactive mass spectrometer (Thermo Fisher Scientific). Compounds were identified using Tracefinder 4.1 (Thermo Scientific), comparing the exact mass and the retention time against an in-house compound database created with authentic standards.

### Western blotting

HAP1-ΔSHMT2/WT and MDA-MB-231-ΔSHMT2 cells were seeded in 60 mm dishes at 350,000 cells/dish and 850,000 cells/dish, respectively. Cells were stimulated the following day with combinations of 1 mM formate, 50 nM rapamycin, 1 μM SHIN1 (MedChemExpress), 50 μM L-alanosine (Santa Cruz Biotechnology) and HT as indicated. Cells were washed twice in ice-cold PBS, scraped and lysed in cold RIPA buffer (ThermoScientific) containing cOmplete phosphatase and protease inhibitors. Total protein concentration was determined by DC protein assay (Bio-Rad), and equal amount of proteins was separated using precast 3–8% or 4–12% NuPage gels (ThermoScientific) or 15% SDS-PAGE gels (for eIF4EBP1 identification) and transferred to nitrocellulose using an Invitrogen XCell II Blot Module. The membranes were incubated overnight at 4 °C using the following primary antibodies at 1:1000: phospho S6-RP Ser235/236 (#4858), total S6-RP (#2217), phospho CAD Ser1859 (#12662), total CAD (#93925), phospho p70 S6K T389 (#9234), total p70 S6K (#2708), 4E-BP1 (#9644) (Cell Signalling Technologies) and β-tubulin (T4026). Secondary antibodies donkey anti-mouse 800CW and goat anti-rabbit IgG (H+L) Alexa Fluor 680 (Li-COR Biosciences and Thermofisher, respectively) were incubated at 1:5000 dilution. Immunoblots were analysed and protein densities quantified using an Odyssey CLx imager and Image Studio Lite software (Li-COR Biosciences).

### Statistics

Technical replicates were used to calculate statistics in the phosphoproteomics analysis. Biological replicates were used to calculate statistics in the immunoblots metabolomics analyses. Statistical significances were calculated using two-tailed and unequal variance *t* test otherwise indicated.

## Results

### Theoretical hypothesis

**Fig. 1:**
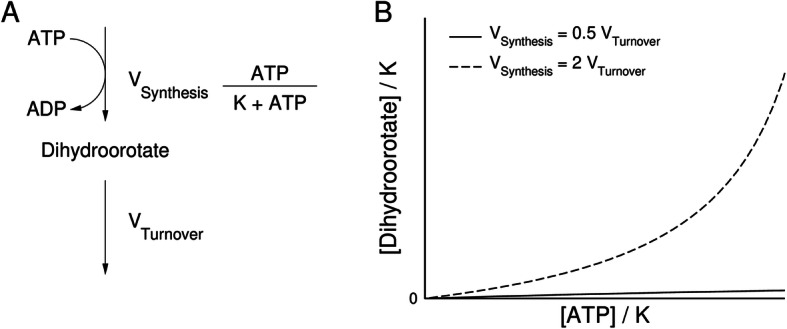
Theoretical model. **a** Schematic representation of the ATP-dependent synthesis of dihydroorotate and its turnover. **b** Expected dependency of the dihydroorotate concentration as a function of the ATP concentration for two different scenarios

To investigate how formate increases pyrimidine synthesis, we developed a hypothesis based on formate increasing ATP levels [1]. We analysed a theoretical model accounting for the synthesis and turnover of the pyrimidine precursor dihydroorotate (Fig. 1a). The synthesis of dihydroorotate has been simplified, for the purpose of illustration, to a reaction following a Michaelis-Menten equation between the reaction rate and the concentration of ATP and a constant rate of turnover. When the maximum rate of dihydroorotate synthesis is less than that of turnover, then the concentration of dihydroorotate changes very little with increasing ATP concentration (Fig. 1b). In contrast, when the maximum rate of dihydroorotate synthesis is higher than that of turnover, the concentration of dihydroorotate exhibits a dramatic increase with increasing ATP concentration.

In order to observe dramatic changes in the concentration of dihydroorotate, it is necessary that the maximum activity of synthesis exceeds that of consumption. Therefore, the increase of dihydroorotate levels upon supplementation of formate can be explained by two hypotheses: (i) either the synthesis of dihydroorotate is higher than its turnover before the addition of formate or (ii) the addition of formate induces an increase in dihydroorotate synthesis relative to turnover.

### Phosphoproteomics reveals CAD phosphorylation

Protein phosphorylation can change the activity of metabolic enzymes providing a biochemical mechanism underpinning the second hypothesis that the addition of formate induces an increase in dihydroorotate synthesis relative to turnover (ii). We previously reported that formate induces changes in phosphorylation levels of AMPK and its downstream targets [1]. Here we used an unbiased approach to identify additional phosphorylation events induced by formate.

The first step in the mitochondrial serine catabolism to formate is catalysed by the mitochondrial serine hydroxymethyltransferase (SHMT2). HAP1 cells genetically engineered to inactivate *SHMT2* (HAP1-ΔSHMT2) exhibit a reduction in formate production relative to the parental cell line (HAP1-WT) [1]. Consequently, HAP1-ΔSHMT2 cells have lower levels of purines and pyrimidines than HAP1-WT cells, a deficiency that can be rescued by supplementation of 1 mM formate in the culture medium [1]. Therefore, HAP1-ΔSHMT2 cells provide a model to investigate phosphorylation events induced by formate supplementation.

**Fig. 2:**
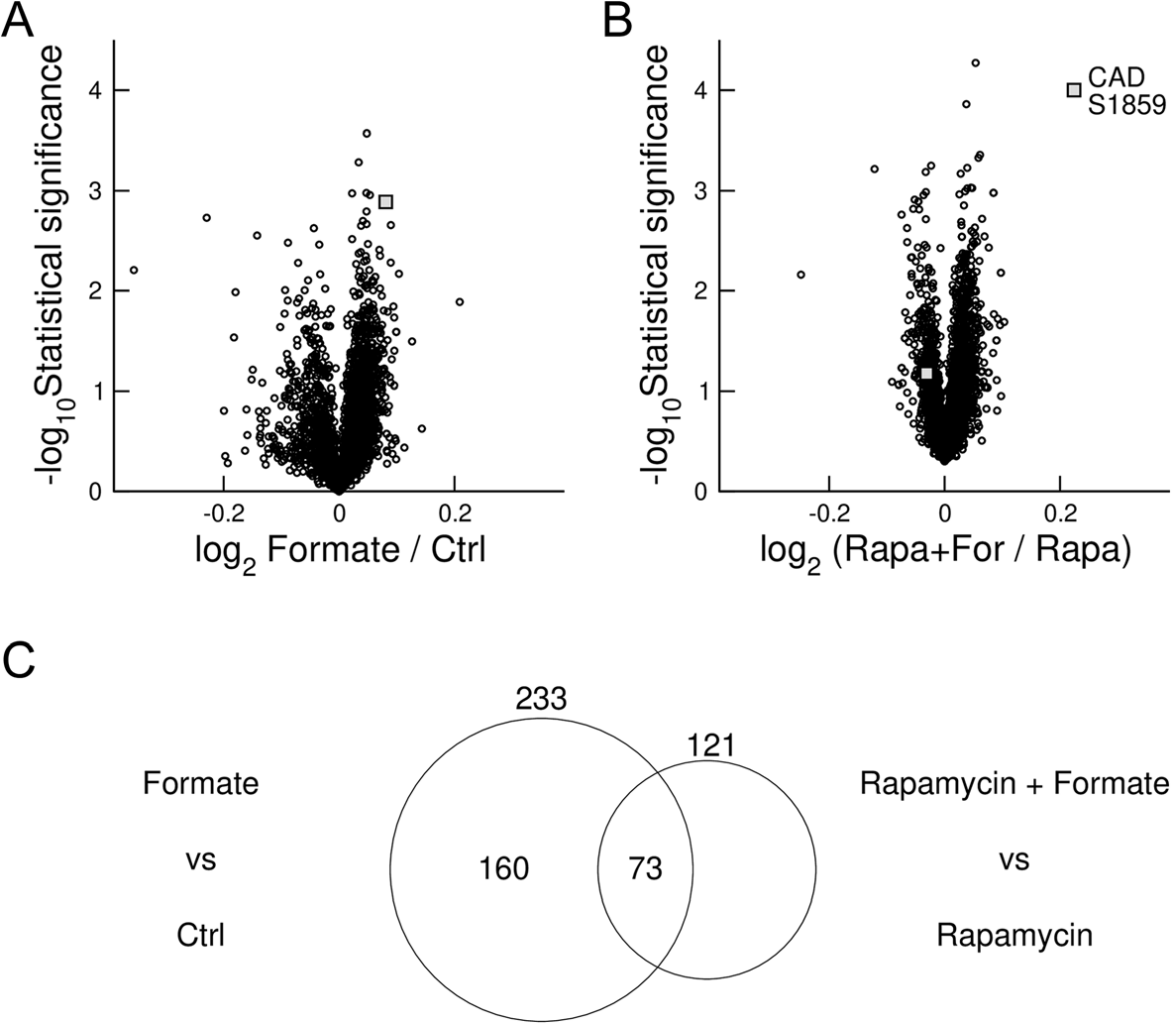
Phospho-proteomic analysis. **a** Volcano plot reporting the log_-_ratio of phosphopeptide quantifications in HAP1-ΔSHMT2 cells supplemented with formate (formate) relative to HAP1-ΔSHMT2 cells (Ctrl), together with the associated statistical significance. **b** Volcano plot reporting the log_-_ratio of phosphopeptide quantifications in HAP1-ΔSHMT2 cells supplemented with formate and treated with rapamycin (Rapa+For) relative to HAP1-ΔSHMT2 cells treated with rapamycin (Rapa), together with the associated statistical significance. Each symbol represents a phosphorylation site. The statistical analysis was conducted on 3 technical replicates from 1 experiment, and the significance was calculated using an unequal variance two-tailed *t* test. **c** Venn diagram highlighting the overlap and non-overlap of significant changes in the absence and under treatment with rapamycin

To investigate the impact of formate supplementation on protein phosphorylation in an unbiased manner, we performed a phospho-proteome analysis of HAP1-ΔSHMT2 cells and HAP1-ΔSHMT2 cells supplemented with formate (Additional file 1). We identified phosphorylation of CAD at Ser1859 as one of the phosphorylation sites that was significantly increased in HAP1-ΔSHMT2 cells supplemented with formate relative to control cells (Fig. 2a).

Phosphorylation of CAD at Ser1859 is known to be regulated by the mTORC1/S6K1 pathway [3, 4]. To investigate whether the formate induced increase in CAD phosphorylation was mediated by the mTORC1 signalling, we analysed the phospho-proteome of HAP1-ΔSHMT2 cells treated with formate in the presence of the allosteric mTORC1 inhibitor rapamycin (Additional file 2). Strikingly, more than half of the formate induced changes in phosphorylation levels in HAP1-ΔSHMT2 cells were abrogated upon rapamycin treatment (Fig. 2c), including the phosphorylation of CAD at Ser1859 (Fig. 2b). These results indicate that the mTORC1 pathway is a major facilitator of the intracellular signalling induced by formate.

### CAD phosphorylation is mediated by mTORC1

**Fig. 3:**
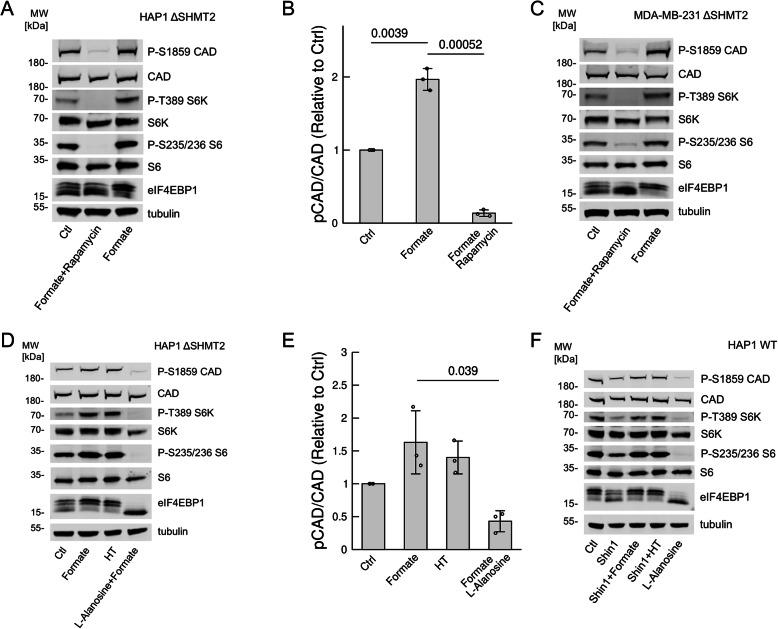
**a** Representative immunoblotting in HAP1-ΔSHMT2 cells under the indicated conditions. **b** Quantifications of CAD phosphorylation related to **a**. **c** Immunoblotting in MDA-MB-231-ΔSHMT2 cells under the same conditions. **d** Representative immunoblotting in HAP1-ΔSHMT2 cells under the indicated conditions. **e** Quantifications of CAD phosphorylation related to **d**. **f** Immunoblotting in HAP1-WT cells under the indicated conditions. Doses: 1 mM formate, 50 nM rapamycin, 50 μM L-alanosine, 1 μM SHIN1 and 16 μM thymidine + 100 μM hypoxanthine (HT). Bars represent the average of 3 independent experiments, error bars the standard deviation and symbols data from the individual experiments. The reported *p* values were calculated using a one-sample *t* test (Ctrl vs formate) or a two-samples *t* test with unequal variance and two-tailes (formate vs formate + rapamycin, formate vs formate + alanosine)

To validate the phospho-proteomics profiling, we performed immunoblotting of CAD and CAD phosphorylated at Ser1859 in HAP1-ΔSHMT2 cells. The immunoblots corroborated the significant induction of CAD phosphorylation at Ser1859 by formate supplementation (Fig. 3a, b). We also immunoblotted for other known targets of mTORC1. Formate supplementation-induced ribosomal protein S6 kinase beta-1 (S6K1) phosphorylation at T389, ribosomal protein S6 phosphorylation at Ser235/236 and the mobility band shift of eukaryote Translation Initiation Factor 4EB Binding Protein (eIF4EBP1) (Fig. 3a), canonical events associated with the activation of mTORC1 signalling. All of these events were reverted by treatment with the mTORC1 inhibitor rapamycin (Fig. 3a). Similar results were also observed in the breast cancer cell MDA-MB-231 with genetic inactivation of SHMT2 (Fig. 3c). These data indicate that formate induces CAD phosphorylation through the activation of mTORC1.

### Formate induction of mTORC1 is dependent on purines

It has been previously shown that formate availability modulates purine levels [1] and that purine levels modulate mTORC1 signalling [8, 9]. From this evidence, we hypothesized that formate induces mTORC1 signalling and CAD phosphorylation via purine synthesis. In agreement with this hypothesis, the purine salvage metabolite hypoxanthine was able to induce an increase in mTORC1 signalling and CAD phosphorylation in HAP1-ΔSHMT2 cells (Fig. 3d,e). Similarly, inhibition of endogenous levels of formate by treating HAP1 WT cells with the SHMT1/2 inhibitor SHIN1 [10] suppressed mTORC1 signalling, which could be reverted by both formate and hypoxanthine (Fig. 3f). In contrast, treatment with the purine synthesis inhibitor L-alanosine [11] abrogated the formate-dependent induction of mTORC1 signalling and CAD phosphorylation in HAP1-ΔSHMT2 cells (Fig. 3d, e). Furthermore, L-alanosine caused a reduction of mTORC1 signalling and CAD phosphorylation in the HAP1 WT cells, which have endogenous formate production (Fig. 3f).

### Metabolic profiling up to 6 h

**Fig. 4:**
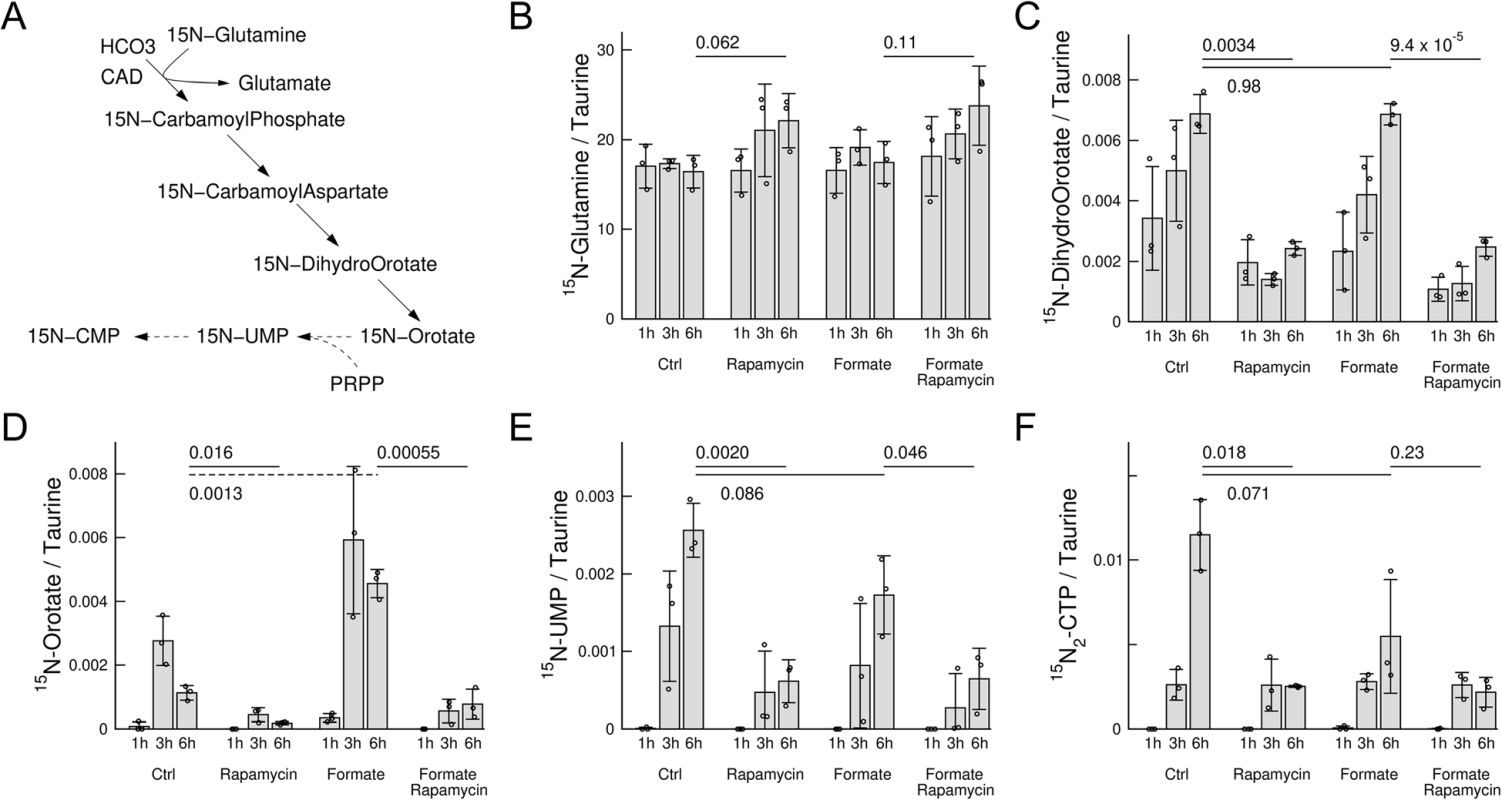
Incorporation of ^15^N into pyrimidines. **a** Schematic representation of the pathway of incorporation of ^15^N from [^15^N-amide]-glutamine into pyrimidine precursors and pyrimidines. Dashed lines indicate multiple chemical transformations. **b**–**f** Peak areas of ^15^N pyrimidine precursors and pyrimidines in HAP1-ΔSHMT2 cells at different times and under different conditions. Quantifications are done relative to the Taurine peak area. Bars represent the mean and error bars the standard deviation from 3 independent experiments, each represented by the symbols. The *p* values shown were obtained using a *t* test with two tails and unequal variance

To investigate the impact of these phosphorylation events upon pyrimidine synthesis, we performed [^15^N-amide]-glutamine tracing experiments. The incorporation of the ^15^N into intracellular metabolites was quantified using mass spectrometry. To test the dependencies on formate and mTORC1, we profiled HAP1-ΔSHMT2 cells untreated, treated with rapamycin, supplemented with formate and supplemented with both formate and rapamycin. The levels of intracellular [^15^N-amide]-glutamine were not significantly differently across these conditions (Fig. 4b).

First, we focused our attention on pyrimidine synthesis (Fig. 4a). In untreated cells, there was an evident increase in the amount of ^15^N-dihyroorotate from 1 to 6 h (Fig. 4c, Ctrl). However, this increase was significantly reduced by treatment with rapamycin. In cells treated with formate, there was a similar rate of incorporation of ^15^N into dihydroorotate as in the untreated cells, which was also significantly reduced upon treatment with rapamycin (Fig. 4c). A similar pattern was also observed when comparing the levels of the downstream pyrimidine precursor orotate and the pyrimidines UMP and CTP in untreated cells and cells treated with rapamycin (Fig. 4d–f).

Based on the bottleneck model in Fig. 1, these data indicate that the maximum activity of dihydroorotate synthesis does not exceed the maximum activity of dihydroorotate turnover within the first 6 h. In contrast, formate significantly increases the levels of ^15^N-orotate at 6 h, indicating that the maximum activity of orotate synthesis exceeds the maximum activity of orotate turnover within the first 6 h.

**Fig. 5:**
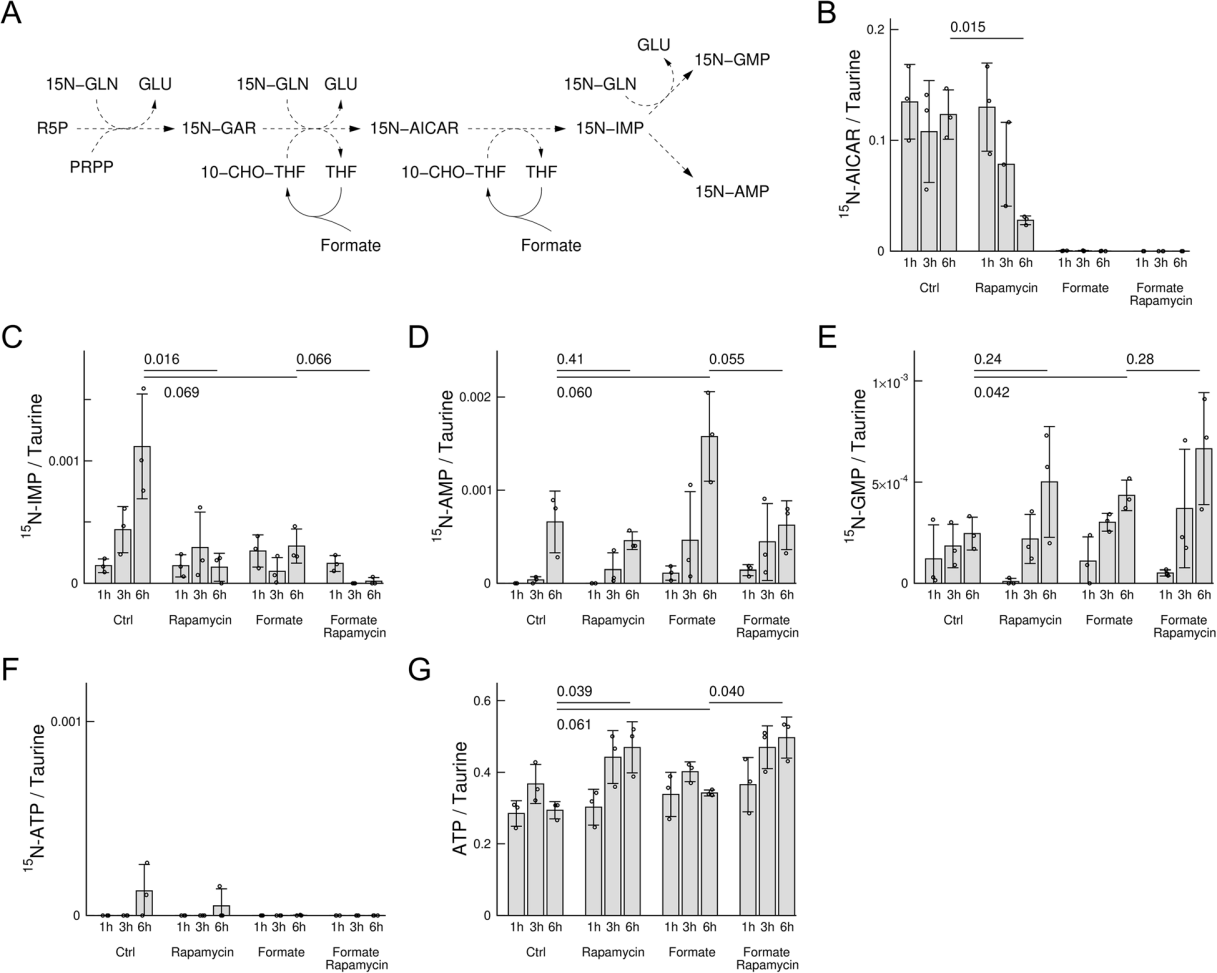
Incorporation of ^15^N into purines. **a** Schematic representation of the pathway of incorporation of ^15^N from [^15^N-amide]-glutamine into purine precursors and purines. **b**–**f** Peak areas of ^15^N purine precursors and purines in HAP1-ΔSHMT2 cells at different times and under different conditions. **g** Total ATP levels (^14^N+^15^N). Quantifications are done relative to the Taurine peak area. Bars represent the mean and error bars the standard deviation from 3 independent experiments, each represented by the symbols. The *p* values shown were obtained using a *t* test with two tails and unequal variance

Next, we focused on purine synthesis metabolites 5-aminoimidazole-4-carboxamide ribotide (AICAR), inosine monophosphate (IMP), adenosine monophosphate (AMP) and guanosine monophosphate (GMP) (Fig. 5a). In untreated cells, there was no significant change in the amount of intracellular ^15^N-AICAR, indicating that the rate of AICAR synthesis is rapid and does not change during this time window (Fig. 5b). In contrast, rapamycin caused a significant depletion of ^15^N-AICAR relative to untreated cells (Fig. 5b). Formate supplementation reduced ^15^N-AICAR to undetectable levels independently of rapamycin treatment (Fig. 5b). For IMP, we observed a similar picture (Fig. 5c), except for a significant time-dependent increase of ^15^N-IMP in untreated cells. Both rapamycin treatment and formate supplementation caused a significant decrease of ^15^N-IMP, as observed for ^15^N-AICAR. Finally, the levels of ^15^N-AMP and ^15^N-GMP were significantly increased by formate independently of rapamycin treatment (Fig. 5d, e, Ctrl vs formate). This result together with the depletion of ^15^N-AICAR and ^15^N-IMP relative to untreated cells suggests an increase in the turnover rate of purine precursors towards the synthesis of purines.

### Metabolic profiling at 24 h

**Fig. 6:**
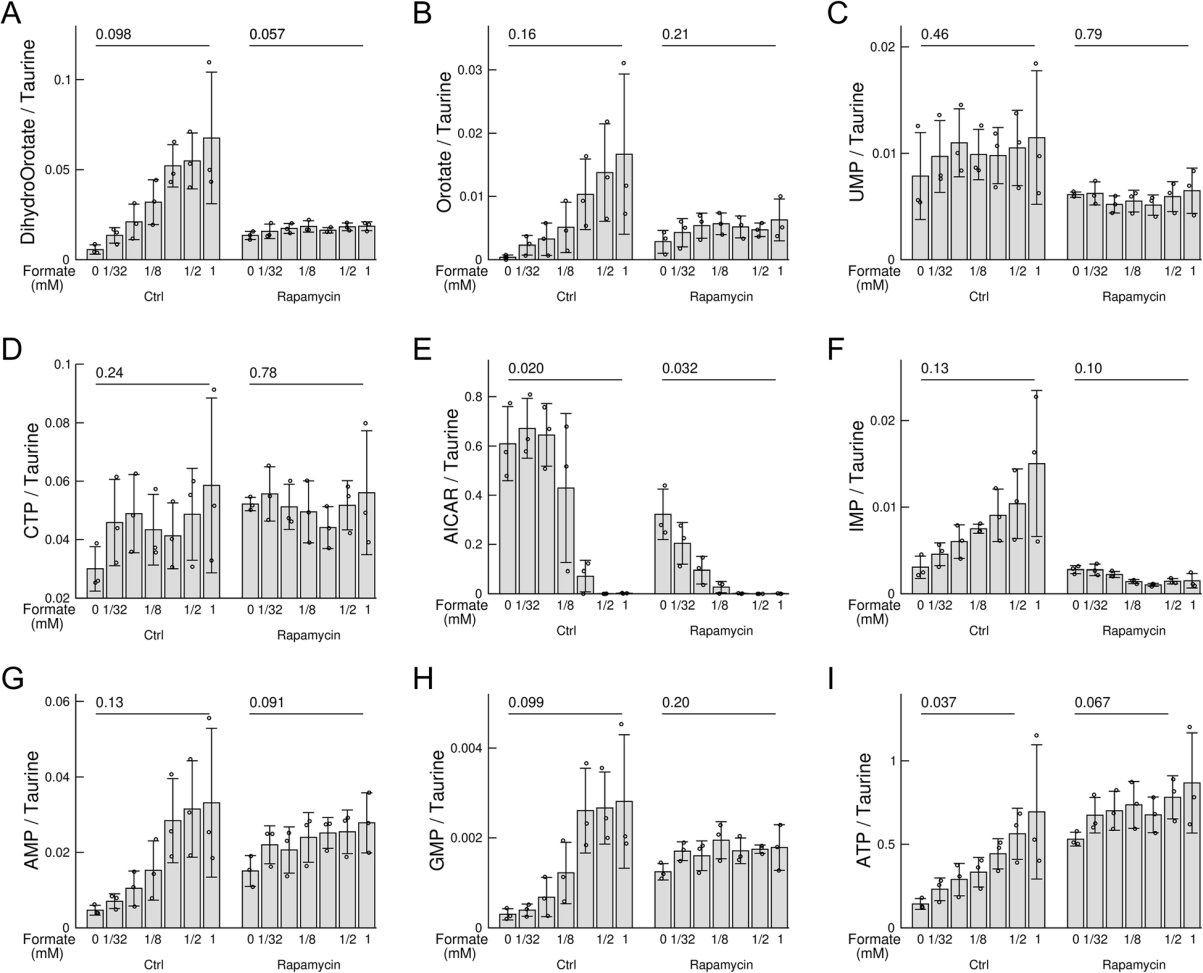
Levels of pyrimidines and purines at 24 h. **a**–**i** Peak areas of the reported metabolites in HAP1-ΔSHMT2 cells supplemented with different concentrations of formate, without or with rapamycin treatment. Quantifications are done relative to the Taurine peak area. Bars represent the mean and error bars the standard deviation from 3 independent experiments, each represented by the symbols. The *p* values shown were obtained using a *t* test with two tails and unequal variance

To investigate the effects of rapamycin and formate on HAP1-ΔSHMT2 cells after longer treatment, we analysed intracellular metabolite levels after 24 h of culture using mass spectrometry. As previously shown [1], most pyrimidines, purines and their precursors increased with increasing the concentration of formate (Fig. 6a–i). In the case of the pyrimidines UMP and CTP, the formate-dependent increase was not as pronounced, but there was no evidence of a decreased level at 24 h.

In contrast, formate had no effect on the levels of most pyrimidines, purines and their precursors when cells were treated with rapamycin. This observation agrees with the finding of rapamycin repression of ^15^N incorporation into dihydroorotate and orotate in the first 6 h, and the repression of CAD-Ser1859 phosphorylation by rapamycin (Fig. 6a–i and Figs. 2 and Fig. 3).

There is one noted exception. Formate induced a decrease rather than an increase of AICAR levels, particularly at the highest formate concentrations (Fig. 6e). The same behaviour was previously observed for HAP1 cells with inactivation of both the cytosolic and mitochondrial pathways of serine one-carbon metabolism [1]. Furthermore, these observations were not changed by rapamycin treatment (Fig. 6e).

Based on the bottleneck model of Fig. 1, the increase in both dihydroorotate and orotate at 24 h (Fig. 6a, b) indicates that at this time point, the maximum activity of both dihydroorotate and orotate synthesis exceeds the maximum activity of their turnover. We noted that this was true for orotate but not for dihydroorotate at 6 h (Fig. 4c, d). This difference suggests that there may be an early increase of CAD activity that is further increased later on, finally leading to the accumulation of all pyrimidine precursors. One possibility for the later increase is the allosteric induction of CAD activity by ATP [2]. There is no ^13^C incorporation into ATP up to 6 h (Fig. 5f), and ATP levels are not changed by formate during the first 6 h (Fig. 5g). In contrast, ATP levels are increased significantly at 24 h (Fig. 6i). Further work is required to determine whether the allosteric regulation of CAD by ATP is indeed playing a role.

## Discussion

Our work shows that the formate induction of pyrimidine synthesis is mediated by the mTORC1/S6K1-dependent phosphorylation of CAD at Ser1859. We also provide evidence that these phosphorylation events are just the tip of the iceberg. More than half of the protein phosphorylation changes induced by formate supplementation are suppressed by treatment with rapamycin.

Formate induction of mTORC1 signalling is consistent with the formate-dependent increase of purine synthesis and the previously reported mTORC1 activation by purine nucleotides [8, 9]. To close the loop, it has been also reported that mTORC1 induces mitochondrial serine catabolism to formate and purine synthesis through activation of the transcription factor ATF4 [12]. Mechanistically, mTORC1 activates ATF4, which then induces the expression of mitochondrial methylene tetrahydrofolate dehydrogenase (MTHFD2), the second step in the mitochondrial serine catabolism to formate. Therefore, our data has unveiled that formate metabolism and mTORC1 signalling are coupled through a positive feedback loop.

The extrapolation of these observations to mammalian physiology requires further work. There are mammalian tissues with active purine synthesis, where the formate-dependent regulation of mTORC1 signalling may play a role. The inactivation of mitochondrial formate production genes carries as a consequence neurodevelopmental defects and embryonic lethality, which can be rescued by sodium formate supplementation [13, 14]. mTORC1 activators with a specific activity in nervous system cells have been recently developed [15]. It will be interesting to see whether mTORC1 activators could rescue the embryonic lethality caused by genetic inactivation of mitochondrial formate production.

Tumour metabolism is also characterized by an induction of mitochondrial formate production genes and purine synthesis [1]. In turn, genetic inactivation of mitochondrial formate production genes reduces tumour growth in subcutaneous xenograft and leukaemia models [16, 17]. However, it is not clear what specific downstream effect or combination of effects (purine synthesis, mTORC1 activation, pyrimidine synthesis) is responsible for the requirement of mitochondrial formate metabolism for enhanced cancerous growth.

Finally, the relationship between mTORC1 signalling and formate availability requires further investigation from the nutritional point of view. Currently, we have a very poor understanding of the nutritional demand of formate or one-carbon units in general [18, 19]. Understanding the relationship between the availability of formate and mTORC1 signalling in normal physiology could have important implications for the management of optimal growth in humans and mammals.

## Conclusion

Formate activates mTORC1 and induces pyrimidine synthesis through mTORC1-dependent phosphorylation of CAD at Ser1859.

## Supplementary information

**Additional file 1.** Phosphoproteomics quantifications of HAP1-ΔSHMT2 cells supplemented with formate and controls.

**Additional file 2.** Phosphoproteomics quantifications of HAP1-ΔSHMT2 cells supplemented with formate and controls, with rapamycin treatment.

## Data Availability

The raw MS files and search/identification files obtained with MaxQuant are deposited at the ProteomeXchange Consortium (http://proteomecentral.proteomexchange.org/cgi/GetDataset) via the PRIDE partner repository [20] with the project name “Formate-induced phosphor-signalling” and project accession PXD019347.

## Abbreviations

mTORC1: Mechanistic target of rapamycin complex 1
CAD: Carbamoyl phosphate synthetase
S6K1: Ribosomal protein S6 kinase 1
SHMT2: Serine hydroxymethyltransferase, mitochondrial
WT: Wild type
eIF4EBP1: Eukaryote translation initiation factor 4EB-binding protein
MTHFD2: Methylene tetrahydrofolate dehydrogenase, mitochondrial
ATF4: Activating transcription factor 4
AICAR: 5-Aminoimidazole-4-carboxamide ribotide
IMP: Inosine monophosphate
AMP: Adenosine monophosphate
GMP: Guanosine monophosphate
ATP: Adenosine triphosphate
